# Nonmedical prescription drug users in private vs. public substance abuse treatment: a cross sectional comparison of demographic and HIV risk behavior profiles

**DOI:** 10.1186/1747-597X-9-9

**Published:** 2014-02-04

**Authors:** Catherine L O’Grady, Hilary L Surratt, Steven P Kurtz, Maria A Levi-Minzi

**Affiliations:** 1 ARSH: Center for Applied Research on Substance Use and Health Disparities, Nova Southeastern University, 2 NE 40th Street, Suite 404, Miami, FL 33137, USA

**Keywords:** HIV, Substance treatment, Nonmedical prescription drug use

## Abstract

**Background:**

Little is known regarding the demographic and behavioral characteristics of nonmedical prescription drug users (NMPDUs) entering substance abuse treatment settings, and information on the HIV-related risk profiles of NMPDUs is especially lacking. Participation in substance abuse treatment provides a critical opportunity for HIV prevention and intervention, but successful initiatives will require services appropriately tailored for the needs of NMPDUs.

**Methods:**

This paper compares the HIV risk profiles of NMPDUs in public (n = 246) and private (n = 249) treatment facilities. Participants included in the analysis reported five or more recent episodes of nonmedical prescription drug use, a prior HIV negative test result, and current enrollment in a substance abuse treatment facility. A standardized questionnaire was administered by trained interviewers with questions about demographics, HIV risk, and substance use.

**Results:**

Private treatment clients were more likely to be non-Hispanic White, younger, and opioid and heroin users. Injection drug use was higher among private treatment clients, whereas public clients reported higher likelihood of trading or selling sex. Public treatment clients reported higher rates of HIV testing and availability at their treatment facilities compared to private clients.

**Conclusions:**

Findings suggest differing demographics, substance use patterns, profiles of HIV risk and access to HIV testing between the two treatment samples. Population tailored HIV interventions, and increased access to HIV testing in both public and private substance treatment centers, appear to be warranted.

## Background

### Treatment of prescription drug abuse and dependence

The nonmedical use of prescription drugs (NMUPD) is an escalating problem in the United States [1-3] and, by population prevalence, appears to have overtaken most illicit substances. NMUPD now exceeds all categories of abused substances, with the exception of marijuana [4,5]. Two of the most commonly prescribed classes of prescription drugs with abuse potential are benzodiazepines, or central nervous system depressants, and opioids [6]. Although benzodiazepines are typically prescribed as short term therapy for anxiety or sleep disorders [6] and opioids are indicated to control acute or chronic pain [6,7], many people use these medications in nonmedical ways or without a valid prescription. Long term or nonmedical use of opioids or benzodiazepines, such as taking larger doses of the medication, can result in tolerance and/or dependence [6,8]; large doses of opioids, or combined use of opioids and another depressant such as alcohol, can lead to respiratory depression or death [6]. Moreover, combining benzodiazepines with other substances such as alcohol have similar outcomes [6]. NMUPD results in more emergency room visits than any illicit drug [3], and is involved in more deaths than heroin or cocaine [3,8].

In addition to heightened misuse, estimates of treatment admission rates for primary use of opiates other than heroin have increased substantially from 2000 to 2010 across the United States [9]. Despite the increasing prevalence of nonmedical prescription drug users (NMPDUs) in substance abuse treatment facilities, the literature describing these populations and the efficacy of treatment is sparse [2,10]. Moreover, accurate estimates of the numbers of NMPDUs in treatment facilities are not available due to a lack of data characterizing specific prescription medication use and a lack of studies that include both private and public treatment facilities [11]. Health and social risk profiles of treatment seeking NMPDUs are needed, as these individuals are likely to differ from other substance users in terms of specific characteristics that could impact treatment services needs, such as underlying medical conditions and demographics [10,12-14].

### HIV risk and NMPDU

A comprehensive profile of treatment-based NMPDUs would include information on HIV behavioral risks, including injection drug use and high risk sexual behaviors. Definitive information describing the injection of prescription medications is lacking. Some studies have found that injection drug use is more common among users of certain prescription opioids such as morphine [15,16] and extended release oxycodone [17,18]. Research has also suggested that the injection of prescription medications is a result of a progression through other, more conventional routes, and is therefore practiced by experienced users [17-19]. Perhaps contradictorily, other studies have associated younger age with a higher likelihood of prescription opioid injection [17,19,20].

Sexual risk behaviors include sex without a condom, trading or selling sex, sex with an injection drug user, or having an elevated number of sex partners [21]. Although the link between illicit drug use and increased sexual behavior risk is well documented [21-25], there is less information available about NMPDUs and increased sexual risk. Among those misusing opioids and benzodiazepines, studies have found associations with HIV sexual risk among samples of undergraduate university students [26], men who have sex with men [27,28] and oxycodone users [29]. However, information on sexual HIV risk from a large and diverse sample of NMPDUs is not apparent in the literature.

### Substance abuse treatment and HIV

Substance abuse treatment can impact HIV transmission through reductions of both injection drug use [30] and sexual risk behaviors [31]. As substance relapse is a common phenomenon, enrollment in treatment often represents a break from risky behaviors, providing an opportunity to intervene [32]. For injection drug users, needle risk interventions include HIV education focusing on behavioral skills and risk reduction, such as information on using bleach solutions to clean needles [30] or using syringe exchange programs [33]. Sexual risk reduction interventions target both information about HIV and relevant behavioral changes [31]. Testing for HIV during treatment can also reach both injection and non-injection substance users who are unaware of their status [34].

The HIV risk behavior profiles of NMPDUs entering substance abuse treatment have not been well documented. Recent studies suggest that treatment seeking prescription opioid abusers differ from heroin users in terms of demographic and drug use profiles [14] and indicate that sexual risk behaviors are elevated among prescription opioid misusers entering detoxification treatment [29]. Given that the number of treatment admissions is escalating at a national level [9], characterizing the risk profiles and service needs of this population assumes great importance.

In this regard, the primary goal of this paper is to examine and compare the demographic and HIV-related risk behavior profiles of NMPDUs in private and public treatment facilities; public treatment centers are federally, state and/or locally funded while private centers require direct monetary payment or insurance coverage. The funding source for treatment tends to impact client/staff ratios, program size, as well as other key aspects of the treatment environment [35]. Client profiles are also likely to diverge, as many individuals in public treatment centers are referred from the criminal justice system, often paying small or sliding scale fees to attend treatment [36], and presumably are without access to private medical insurance or other financial resources.

Prior surveillance efforts have indicated that HIV testing and prevention services tend to be more widely available in publically-funded treatment programs when compared with privately operated facilities [37,38]. Nevertheless, there is little extant evidence to indicate that this difference reflects the actual risk profiles of the patients being treated. As such, this paper sought to explore differences in NMUPD patterns and HIV risk behavior profiles, between private and public treatment clients for the purposes of understanding whether existing HIV-related services are appropriately matched to meet the needs of the target populations. Background information, demographics, social and HIV risk assessments are necessary for designing effective HIV interventions and educational programs for this growing population of treatment enrollees. By gaining an understanding of the risk profiles of NMPDUs who present in different treatment settings, researchers are equipped to assess the adequacy of current HIV service levels and recommend changes in treatment program policies related to access and uptake of HIV testing and support services.

## Methods

### Study sample

Eligible participants were age 18 or over and reported five or more occasions of NMUPD within the 90 days before entering substance treatment. The larger study from which the sample was drawn (n = 1,629) included six subgroups with different eligibility criteria: participants aged 60 and older (n = 126), stimulant using men who have sex with men (n = 300), methadone maintenance clients (n = 301), street-based drug users (n = 301), publically-funded residential treatment clients (n = 300) and privately-funded residential treatment clients (n = 301). For the current analysis, we included only public and private treatment clients.

Study recruitment procedures varied by program according to their internal policies. In some programs, participants were informed about the study by treatment staff, either by center wide announcements or during intake assessments. Eligibility screening was performed by treatment staff, who informed the researchers of eligible interviewees. In other programs, research staff were permitted to conduct the eligibility screening. All interested participants were screened for eligibility to participate in this study. All participants were assured that their participation in the study was voluntary, and had no impact on their status in their respective treatment programs.

Interviews took place in South Florida residential in-patient treatment centers. Three public treatment centers and three private treatment centers provided interviewees. All treatment centers are well established and admit between 350 to over 1000 clients per year. Following informed consent, face to face confidential structured interviews were conducted in private offices by trained research staff. Interviews lasted about 1-1/2 to 2 hours. After the completion of the interview, participants were compensated with $30. Study protocols and instruments were approved by the University of Delaware (predecessor institution) and Nova Southeastern University Institutional Review Boards, which follow the Helsinki Declaration of 1975.

### Measures

The primary instrument was the Global Appraisal of Individual Needs (GAIN-I) [39], an assessment tool that contains measures of substance use and dependence and sexual risk behaviors. The GAIN-I has been used regularly in substance treatment research [40,41]. In our current survey, questions about the nonmedical use of prescription medications (opioids, benzodiazepines, stimulants, antidepressants and antipsychotics) and routes of administration were added. All substance use questions used a 90 day recall period for days of misuse before entering treatment.

HIV sexual risk measures were assessed through dichotomous questions about behaviors within the past 12 months: having sex with an injection drug user, having two or more sex partners, having unprotected sex, and selling or trading sex. Measures for HIV needle risk were dichotomous questions: a) in the past year, using a needle to inject drugs, reusing a needle, and using someone else’s rinse water, cooker or cotton; and b) in the past 90 days before treatment entry, using a needle to inject a prescription medication.

HIV testing availability in the treatment center was measured by three yes or no questions. If a participant answered “do not know” or “no” when asked if HIV testing was available in the treatment facility, they were not asked the subsequent questions: “have you been offered HIV testing” and “did you participate in HIV testing”.

### Data analysis

Participants who reported a positive HIV test (n = 16) or who did not report a prior HIV test (n = 48) were eliminated from the analysis in order to focus on the assessment of HIV infection risk and access to HIV testing among seronegative individuals.

Primary prescription drug was calculated by determining the medication that the participants reported misusing on the greatest number of days in the 90 days prior to treatment. Primary drug class (opioid or benzodiazepine) was determined by the class of drug used the greatest number of days in the 90 days prior to treatment. Only participants that had a clear primary drug class (opioid or benzodiazepine (n = 495; exclusions = 42)) were included in this analysis.

All analyses were conducted using SPSS version 20. Descriptive statistics characterized the overall profile of the sample and the public and private treatment groups separately. These measures included age, gender, income, education, race/ethnicity, health insurance status and primary prescription substance of misuse.

Bivariate logistic regression models were constructed to examine associations between treatment venue type, demographics and sexual and needle HIV risks and access and uptake of HIV testing. Private treatment clients were the reference group for all analyses. Participants were excluded from specific analyses if data were missing or the participant did not answer. The Ns for each analysis are included in Table 1.

**Table 1:** Public and private treatment clients’ demographics, substance use characteristics, HIV risk behaviors and testing availability

		**Private (n = 249)**	**Public (n = 246)**	**Wald**	**OR (95% CI)**	**p-value**
		**n(%)**	**n(%)**			
Demographics						
	Age (under 30 yrs)	162 (65.1)	109 (44.3)	21.18	0.43 (0.30, 0.61)	<0.01
	Female gender	97 (38.9)	104 (42.3)	0.57	1.15 (0.80,1.64)	0.45
	Has medical insurance^a^	175 (70.6)	76 (31.3)	71.60	0.19 (0.13, 0.28)	<0.01
	Graduated high school	165 (66.3)	114 (46.3)	19.68	0.44 (0.31, 0.63)	<0.01
	Income $1000 or less in the last month^a^	73 (30.5)	126 (53.8)	25.79	2.65 (1.82, 3.87)	<0.01
	Lifetime arrests, mean (sd)	4.01 (7.35)	9.00 (11.29)	27.83	1.09(1.05, 1.12)	<0.01
Race/Ethnicity						<0.01
	Non-hispanic white	207 (83.1)	90 (36.6)	99.68	0.12 (0.08, 0.18)	<0.01
	Non-hispanic black/ African-American	10 (4.0)	42 (17.1)	19.11	4.92 (2.41, 10.05)	<0.01
	Hispanic/Latino	18 (7.2)	101 (41.1)	62.57	8.94 (5.19, 15.38)	<0.01
	Other ethnicity	14 (5.6)	13 (5.3)	0.03	0.94 (0.43, 2.04)	0.87
Primary drug class						
	Opioids	199 (79.9)	131 (53.3)	37.85	0.29 (0.19, 0.43)	<0.01
	Benzodiazepines	50 (20.1)	115 (46.7)	37.85	3.49 (2.35, 5.21)	<0.01
Other substances used						
	Crack cocaine	84 (33.7)	127 (51.6)	16.00	2.10 (1.46, 3.01)	<0.01
	Powder cocaine	135 (54.2)	153 (62.2)	3.23	1.39 (0.97, 1.99)	0.07
	Heroin	82 (32.9)	41 (16.7)	17.00	0.41 (0.27, 0.62)	<0.01
Routes of administration (90 days before treatment)						
	Injected pills^b^	88 (35.3)	42 (18.8)	15.65	0.43 (0.28, 0.65)	<0.01
	Smoked pills^b^	73 (29.3)	42 (18.8)	6.93	0.56 (0.36, 0.86)	0.01
	Snorted pills^b^	175 (70.3)	112 (50.2)	19.52	0.43 (0.29, 0.62)	<0.01
Needle risk (past year)						
	Injected a drug	107 (42.9)	56 (22.8)	22.30	0.39 (0.27, 0.58)	<0.01
	Reused someone’s needle^c^	36 (33.6)	26 (46.4)	2.53	1.71 (0.88, 3.31)	0.11
	Using someone else’s rinse water, cooker or cotton^a,c^	33 (30.8)	24 (43.6)	2.58	1.74 (0.89, 3.40)	0.11
HIV sexual risk behaviors (year before treatment)						
	Sex with an injection drug user^a,d^	55 (23.1)	36 (15.9)	3.84	0.63 (0.39, 1.00)	0.05
	Traded or sold sex^d^	32 (13.4)	63 (27.4)	13.74	2.38 (1.46, 3.81)	<0.01
	Two or more recent sexual partners^d^	143 (59.8)	148 (64.3)	1.01	1.21 (0.83, 1.76)	0.31
	Had sex without a condom or barrier^a,d^	214 (90.3)	186 (80.9)	8.16	0.45 (0.26, 0.78)	<0.01
	HIV testing is available to you in this program^a^	117 (79.1)	199 (95.7)	19.92	5.86 (2.69, 12.74)	<0.01
	Has been offered HIV testing since arrival^a,g^	64 (55.7)	148 (75.1)	12.36	2.41 (1.48, 3.93)	<0.01
	Participated in HIV testing at this program^a,g^	27 (24.3)	117 (60.6)	34.73	4.79 (2.85, 8.06)	<0.01

Note. Private treatment is the reference group. Df =1 for all. ^a^Participants were excluded that did not answer or answered “do not know”. ^b^Early initiates into the study were not included (n = 23). ^c^Participants who had not injected a drug within the last twelve months were not included. ^d^Participants who had not had sex within the last twelve months were not included (n = 26). ^g^Only participants who answered “yes” to the question, “is HIV testing available to you in this program” were included.

## Results

Comparisons of public and private treatment participants are reported in Table 1. Participants were currently enrolled either in public (n =246/495; 49.7%) or private (n =249/495; 50.3%) substance treatment facilities. Public treatment clients were more likely to identify as Latino/Hispanic (41.1% vs. 7.2%) or Black/African-American (17.1% vs. 4.0%) and were older (mean age: 33 vs. 29 for private treatment clients, see Table 1) than their private treatment counterparts. Public treatment clients had been arrested more often than private treatment clients (9 vs. 4 times, see Table 1). More private treatment clients identified as non-Hispanic White (83.1% vs. 36.6%, see Table 1) and reported having health insurance than public treatment clients (70.6% vs. 31.3%, see Table 1). Participants enrolled in public treatment centers reported lower incomes, with 53.8% earning $1000 or less in the past month, compared to 30.5% of private treatment clients (see Table 1), and were less likely to have graduated high school (46.3% vs. 66.3%, see Table 1). The gender breakdown was similar among both private (38.9% female) and public (42.3% female) treatment groups (see Table 1).

Public and private treatment clients differed significantly on the primary prescription drugs that they used most often for nonmedical purposes. Public treatment clients were more likely (46.7%) than private (20.1%) clients to report primary use of benzodiazepines (see Table 1), while primary use of opioids was less likely among public treatment clients (53.3% vs. 79.9%, see Table 1).

Many participants also used illicit substances in addition to misusing prescription medication. The two groups displayed significant differences in the types of substances endorsed. Public treatment clients were more likely to report use of crack cocaine in the 90 days before treatment entry (51.6% vs. 33.7%, see Table 1). Clients who used heroin in the 90 days before treatment were less likely to be enrolled in public treatment clinics (16.7% in public treatment vs. 32.9% in private treatment, see Table 1).

HIV risk behaviors were prevalent among the entire sample, however public and private treatment clients differed on several HIV risk measures. The treatment groups did not differ in the number of recent sex partners or the likelihood of having a primary partner. However, private treatment clients were more likely to report unprotected sex (see Table 1) and having had sex with an injection drug user (see Table 1) in the past year than their public treatment counterparts. Public treatment enrollees were more likely than private treatment clients to endorse recent selling or trading sex for money, drugs or gifts (27.4% vs. 13.4%, see Table 1).

Private treatment clients were more likely than public treatment clients to have injected drugs in the year before entering treatment, to have injected a prescription medication in the last 90 days before treatment, and to have ingested prescription pills through higher risk snorting or smoking routes (see Table 1).

Although private treatment clients reported higher injection-related HIV risk behaviors in the analyses, on average they had previously been tested for HIV fewer times than public treatment clients (4.4 vs. 6.8 times, T-Test: df = 375; t = -3.43, p < 0.01). In addition, the availability and uptake of HIV treatment in substance treatment facilities was less prevalent for private treatment clients. Public treatment clients were more likely to have HIV testing available through their treatment facility than private clients (95.7% vs. 79.1%, see Table 1). Of those who reported HIV testing was available at their facilities, public treatment clients were also more likely to have been offered the testing than were private clients (75.1% vs. 55.7%, see Table 1), and among those who had HIV testing available at their treatment facilities, public treatment clients were more likely to participate in testing (60.6% vs. 24.3%, see Table 1).

## Discussion

In this diverse sample of public and private NMUPD treatment clients, our findings reveal several demographic and socio-economic differences between the two treatment groups. Significant associations with public treatment type were Latino or Black/African-American ethnicity, and having a lower income. These data correspond to recent statistics that suggest Blacks/African-Americans and Hispanic/Latinos have lower median household incomes and are less likely to have health insurance coverage than non-Hispanic Whites [42]. Our findings are generally consistent with prior research in the area of health disparities, including greater unmet need for substance abuse treatment among African Americans and Latinos relative to Whites due to a lack of financial resources [43]. Importantly, prior research has also shown that Whites are more likely to access substance abuse treatment in specialty facilities compared to racial and ethnic minorities [44]. While all of the programs studied here were specialized substance abuse treatment centers, the private programs tended to be smaller in size, with lower staff/client ratios, which likely confers an advantage to these clients in terms of a personalized and focused treatment experience. Although the examination of treatment outcomes was beyond the scope of the present study, our findings nevertheless highlight substantial race/ethnic disparities in access to private substance abuse treatment.

Our results showed that public treatment clients were more likely to be users of crack cocaine than were private treatment clients. Given their lower overall income levels, drug of choice may have been influenced by socioeconomic factors. Prior research has indicated that crack cocaine use tends to be clustered among lower socioeconomic status communities due to its lower costs as compared to other substances such as cocaine [45,46]. In addition, public treatment clients are also more likely to have traded or sold sex (see Table 1). This is likely driven by this elevated prevalence of crack cocaine among public participants, which has long been associated with transactional sex and survival sex work [25,47].

Benzodiazepine users were 3.49 times more likely to be in public treatment as compared to opioid users (see Table 1). We speculate that this finding is tied to the higher prevalence of cocaine use among the public treatment group, as benzodiazepines are reportedly used to come down from stimulants or moderate cocaine withdrawal [48].

Significant associations with private treatment enrollment were younger age, non-Hispanic White ethnicity, having a high school education and having medical insurance. Private facility clients were 2.44 times more likely to report recent heroin use and 3.49 times more likely to have primary prescription opioid misuse. This is not surprising as the two are frequently substituted [49]. It is not unexpected that medical insurance coverage and higher income were associated with private treatment enrollment, given that enrollment in these facilities is based on ability to pay. Similarly, the younger age association with private treatment might also reflect access to parents’ financial resources, and may have been influenced by the Patient Protection and Affordable Care Act of 2010, which extended parents’ insurance health care coverage to their children under the age of 26 [50].

In addition to differences in demographics and substance use profiles, the treatment groups also differed in patterns of HIV risk behavior in the analyses, including route of drug administration and sexual risk behaviors. Although HIV risk behaviors were clearly present in both samples, private treatment clients reported higher levels of several recent risk behaviors in bivariate regressions than public treatment clients, including injection drug use. Because private treatment clients had higher income and resources, our findings appear to diverge from recent research suggesting that transition to injection drug use is economically motivated as a cheaper route of administration [18].

In addition to these differences in risk behaviors, the availability, accessibility and uptake of HIV testing services were reported less frequently by private as opposed to public treatment clients. The higher prevalence of several sexual and needle risk behaviors among private treatment participants is particularly worrisome as they reported fewer prior HIV tests. This resonates with other studies that have documented a higher prevalence of prevention services (such as sexually transmitted infection testing) in public as compared to private substance treatment facilities [30,51]. One recent nationwide study documented that only 27% of private treatment facilities offered HIV testing [52].

### Strengths

This study included large and diverse samples of public and private substance abuse treatment clients. Comparisons of HIV risk behaviors between the two groups are not apparent in prior studies. Other strengths include the focus on NMPDUs, who are underrepresented in the literature and comprise a growing proportion of new treatment admissions. As well, the data were collected using a comprehensive assessment of nonmedical prescription drug use, including both opioids and benzodiazepines.

### Limitations

There are several limitations to this study. First, the results may not be generalizable to larger populations of NMPDUs, as this study was conducted in South Florida and included non-probability samples. In addition, we used a cross sectional design, and as such causal links cannot be established. Further, substance use, sexual behaviors, and HIV status were self-reported data, and may be susceptible to social desirability and/or recall bias. However, these concerns are reduced given the high levels of reports of these behaviors.

## Conclusions

The public and private treatment samples that were included in this study had differing demographics and substance use patterns. HIV risks appeared substantial in both groups, and HIV testing was not easily available to many of the clients in the substance treatment centers. As such, HIV testing should be implemented more universally, and tailored HIV interventions should be introduced.

The CDC recommends that all substance abuse treatment clients receive routine HIV testing that is not contingent on their individual risk factors [53]. Routine testing reaches a broader base of patients as all individuals are tested unless they decline [54]. The value of HIV testing in substance abuse treatment includes its cost effectiveness [53,54] and a likely reduction in risky sexual behaviors after a positive test [55], which could lead to reductions in HIV transmission. In addition to the benefit of reduced HIV transmission, routine HIV testing can lead to quicker linkages with HIV care [53]. Despite these benefits, only a minority of states require HIV testing services to be provided during substance abuse treatment. In those states requiring testing availability, it is usually mandatory only in facilities receiving state funding [56]. In this study, we found that the CDC’s recommendations [53] failed to be fully implemented as many participants, particularly the private treatment clients, were not offered routine HIV testing while in treatment. Both groups of participants had substantial risk factors for HIV and would benefit from the routine testing procedures.

Although HIV testing recommendations include the universal availability to substance treatment clients, our study also points to the need for HIV sexual risk interventions that are tailored to the needs of the clients being served. This is important as the samples of private and public treatment clients differed on both demographic and HIV risk factors. Sexual risk reduction interventions during substance abuse treatment appear to be less consistently successful than interventions to reduce needle risk behaviors [34]. However, such interventions can be efficacious if they are carefully designed. Several studies in treatment settings that implemented interventions ranging from two to five sessions of HIV education and behavioral skills building were associated with significant reductions in HIV sexual risk behaviors [31,57].

It is not immediately clear how often sexual risk reduction interventions are provided in substance abuse treatment facilities, nor how frequently these programs implement evidence-based and carefully designed sessions. A majority of substance treatment facilities in Florida report that they offer such sessions [58], including the treatment centers that participated in this study. However, this does not necessarily indicate the frequency, adherence or attendance of the sessions [56], or whether these interventions are effective among NMPDUs populations.

Understanding the HIV risk profiles of NMUPD treatment enrollees is crucial as their prevalence in treatment settings continues to increase. As best treatment practices for nonmedical use of prescription drugs have not yet been developed [2], this is an ideal time to implement universal HIV testing and population specific educational protocols in substance abuse treatment facilities. This would be particularly beneficial in private substance abuse treatment facilities, where HIV prevention is apparently lacking.

## Competing interests

The authors declare that they have no competing interests.

## Authors’ contributions

The specific contributions of each author are as follows: CO was responsible for the collection of the literature, data analysis, and prepared the first draft of the manuscript. SK and HS were responsible for study design. ML participated in the interviewing and revised the manuscript. All authors provided critical comments on the first draft of the manuscript and approved the final version to be submitted.
